# TET enzymes: double agents in the transposable element–host genome conflict

**DOI:** 10.1186/s13059-016-1124-8

**Published:** 2016-12-20

**Authors:** Patricia Gerdes, Sandra R. Richardson, Geoffrey J. Faulkner

**Affiliations:** 1 Mater Research Institute, University of Queensland, TRI Building, Woolloongabba, QLD 4102 Australia; 2 School of Biomedical Sciences, University of Queensland, Brisbane, QLD 4072 Australia; 3 Queensland Brain Institute, University of Queensland, Brisbane, QLD 4072 Australia

## Abstract

The mouse genome is replete with retrotransposon sequences, from evolutionarily young elements with mutagenic potential that must be controlled, to inactive molecular fossils whose sequences can be domesticated over evolutionary time to benefit the host genome. In an exciting new study, de la Rica and colleagues have uncovered a complex relationship between ten-eleven translocation (TET) proteins and retrotransposons in mouse embryonic stem cells (ESCs), implicating TETs as enhancers in the exaptation and function of retroelement sequences. Furthermore, they have demonstrated that active demethylation of retrotransposons does not correlate with their increased expression in ESCs, calling into question long-held assumptions regarding the importance of DNA demethylation for retrotransposon expression, and revealing novel epigenetic players in retrotransposon control.

Please see related Research article:  http://genomebiology.biomedcentral.com/articles/10.1186/s13059-016-1096-8

## Introduction

Transposable elements (TEs) are dynamic players in genome evolution. Retrotransposons, which mobilize via a “copy-and-paste” mechanism, account for ~40% of the typical mammalian genome. In humans, the only active autonomous retrotransposon is Long Interspersed Element 1 (LINE-1 or L1), while in mice, both L1 and long terminal repeat (LTR) retrotransposons, which resemble retroviruses in structure and function, are presently active [1]. TEs mobilize to ensure their survival and, consequently, must be controlled to protect host genome stability. New TE copies are known to disrupt transcription and can influence gene structure and expression by various mechanisms, which can lead to cancer progression and genetic disease [2]. However, heritable TE insertions are also an ongoing source of genomic diversity that can undergo exaptation over evolutionary time to serve beneficial functions for the host [3]. Thus, it is of key interest to understand the molecular mechanisms by which TEs are controlled and, in some cases, ultimately domesticated.

In a new publication, de la Rica and colleagues have investigated the roles of ten-eleven translocation (TET) enzymes at TE-derived sequences in mouse embryonic stem cells (ESCs) [4]. The pluripotent cells of the early mammalian embryo are the primary milieu for the evolutionary struggle between TEs and the host genome. A genome-wide epigenetic switch in the early mammalian embryo, particularly of global DNA demethylation state, is necessary to activate the programme of embryonic development. This epigenomic “reset” is thought to provide a window of opportunity for retrotransposons to mobilize and create heritable insertions. It has been suggested that DNA methylation of CpG dinucleotides evolved primarily to protect the host against TEs. However, although DNA methylation may be sufficient for TE repression it may not be necessary, as studies report that the loss of DNA methylation is not always followed by a significant increase in retrotransposition [1]. It is likely, therefore, that multiple silencing mechanisms act in concert to control retrotransposon activity in pluripotent cells.

Despite decades of study, the essential principles of the reprogramming process during embryogenesis have not been completely resolved. An active demethylation mechanism involving the TET enzymes has recently been uncovered, overturning the perception that DNA methylation can be erased only passively, upon DNA replication. TET enzymes function through oxidation of 5-methylcytosine (5mC) to 5-hydroxymethylcytosine (5hmC), and further to 5-formylcytosine (5fC) and 5-carboxycytosine (5caC), which can be replaced with unmodified cytosine by base excision repair (BER) [5].

Given the enrichment of 5hmC in mouse ESCs [6], and the evolutionary drive for L1s to mobilize in pluripotent embryonic cells, it stands to reason that active demethylation by TET proteins could act as an on-switch in the control of early embryonic retrotransposition. The publication from de la Rica and colleagues [4] reveals unexpectedly complex scenarios for TET-mediated TE regulation, probably shaped by ongoing evolutionary conflict at the host–retrotransposon interface. Importantly, their results shed light on the importance of DNA methylation relative to other epigenetic mechanisms for TE control in pluripotent cells.

## TET enzymes—multiplayers in TE regulation

### TET enzymes implicated in TE-derived enhancer function

Noting that the field lacked a comprehensive analysis of TET interaction with TE sequences, de la Rica and colleagues [4] mined ChIP-seq data to determine the distribution of TET1 peaks at distinct TE classes. Their analysis revealed significant enrichment of TET1 at L1s and several types of LTR retrotransposons, suggesting that TET1 may have a widespread role in TE regulation. Unexpectedly, they discovered that co-occupancy of TE-derived TET1 binding sites was not universal, but varied depending on TE class. The authors speculated on the reasons for differential co-occupancy at these sites and, indeed, set the stage for future studies to elucidate the molecular basis and functional consequences of the interactions between TET1, other epigenetic factors, and particular TE sequences.

De la Rica and colleagues [4] also observed that TET1 peaks at LTR elements were associated with active enhancer marks, as well as the pluripotency factors NANOG, OCT4, and SOX2 (collectively referred to as NOS). This observation is consistent with the occupied TE sequences acting as enhancers, and led to the hypothesis that TET proteins have a role in TE-derived enhancer function, important for ESC gene expression networks. This theory was further supported by the discovery of interactions between those TE sequences and gene promoters and bidirectional enhancer RNAs generated from such TET-bound enhancers. Indeed, analysis of 5mC and 5hmC levels in *Tet2* knockout mouse ESCs revealed a reduction in 5hmC and an increase in 5mC at NOS-bound TE sequences. Thus, the authors concluded that TET binding and demethylation at particular TE classes acts in concert with NOS factors to maintain expression of a subset of genes in ESCs. Future studies will no doubt shed light on the functional importance of the specific gene–enhancer interactions identified here.

### For L1, demethylation is not equal to expression

De la Rica and colleagues [4] next undertook a detailed examination of TET occupancy at L1 elements in ESCs, which revealed that TET proteins preferentially bind to and participate in active demethylation of full-length, evolutionarily young L1s, but not older, inactive subfamilies. This result raised the question of whether TETs are directly responsible for the demethylation and activation of L1 promoters in ESCs. Unexpectedly, depletion of TET1 and TET2 and a resultant increase in L1 methylation had no effect on L1 expression levels, indicating that DNA methylation status may not be the most important epigenetic determinant of L1 expression in ESCs.

Indeed, further analysis revealed that the 5′ UTRs of young L1s are enriched for the co-repressor complex SIN3A. Remarkably, de la Rica and colleagues here showed for the first time that SIN3A might be involved in TE regulation in mouse ESCs as well as in human ESCs. SIN3A is likely to counter the effect of DNA demethylation of L1 elements by acting as a transcriptional repressor. Thus, TET enzymes may not only be positive regulators of L1 expression, but may instead have a dual role in TE regulation by recruiting SIN3A to demethylated L1 elements. This finding is, therefore, an additional indication for the involvement of multiple layers of regulation in controlling L1 expression in ESCs. It remains to be determined whether similarly layered regulation exists in non-embryonic cell types with high levels of 5hmC and which support high levels of L1 activity (e.g. neurons [7]), and it is notable that L1 RNA expression is only the first step in the generation of a new L1 insertion. Ultimately, the relative importance in controlling mutagenic L1 activity of DNA methylation, the co-repressor SIN3A, and other epigenetic factors will need to be assessed by examining their impact on the accumulation of new L1 insertions in vivo, perhaps by applying targeted sequencing approaches to rodent models. Indeed, the advent of single-cell genomics raises the possibility of identifying new L1 insertions in mouse pre-implantation embryos from genetic backgrounds of interest.

### A new player in an evolutionary arms race

Overall, the results of de la Rica and colleagues [4] implicate TET enzymes in an ongoing evolutionary arms race wherein host defence mechanisms continuously evolve to target expanding TE subfamilies. Strikingly, such conflicts appear to be occurring in both the human and mouse genome, providing an intriguing example of convergent evolution. It was previously shown that evolutionarily old L1 subfamilies are repressed by KAP1 (also known as TRIM28). KAP1 is recruited to the immobile human L1 subfamilies L1PA3, L1PA4, L1PA5, and L1PA6 and is involved in depositing the repressive histone mark H3K9me3 [8]. Species-specific Krüppel-associated box domain-containing zinc finger proteins (KRAB-ZFPs) are also part of this mechanism and were found to recognize and silence L1 [9, 10]. Interestingly, the L1PA3 subfamily escaped silencing through ZNF93 by removal of the ZNF93-binding site and it was suggested that TEs and ZFPs effectively compete in an evolutionary arms race in which KRAB-ZFPs evolve to suppress newly developed TE classes, and this is followed by mutations in these TE classes to avoid this repression [9].

**Fig. 1:**
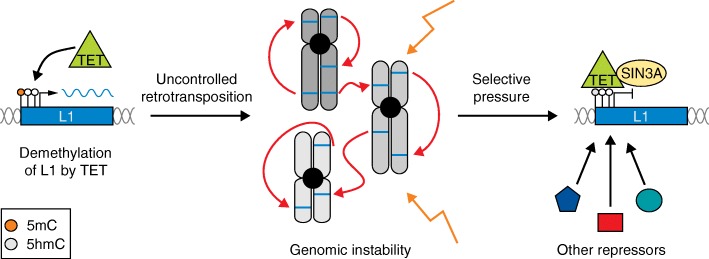
Evolution of TET-mediated repression of L1 elements. L1 retrotransposons bound by TET enzymes (*green triangles*) could become demethylated (*left*), which in turn would result in L1 expression (L1 mRNA indicated in *blue*). Consequently, uncontrolled L1 expansion can lead to genomic instability due to disruption of gene function and creation of DNA double-strand breaks (*centre*). Therefore, selective pressure could have led to TET proteins recruiting other repressors, such as SIN3A (*yellow oval*), to ensure L1 repression and maintain genomic stability (*right*). *5mC* 5-methylcytosine, *5hmC* 5-hydroxymethylcytosine, *L1* Long Interspersed Element 1, *TET* ten-eleven translocation

TET proteins might have become another part of this repression/escape cycle by providing repression for TEs that have escaped the KRAB-ZFP/KAP1 silencing machinery. Mutations in L1 elements might have generated conditions that allow for TET binding leading to DNA demethylation. However, the selective pressure to ensure genome stability may also have led to the evolution of TET-dependent host-silencing strategies to control L1 expression, especially during early embryonic development (Fig. 1). In the long run, as a particular TE class is “defeated” in ESCs by silencing and, ultimately, the accumulation of mutations, it no longer poses a threat to genome integrity and its sequences may undergo exaptation by the host genome, for example, as enhancers or promoters of particular gene expression programmes [3].

## Conclusions

Overall, through integrated genome-wide analyses, de la Rica and colleagues [4] have shown that although retrotransposons are actively demethylated by TET enzymes in ESCs, this does not necessarily equate to transcriptional activation. This result requires a thoughtful re-examination of the widely-held assumption that methylation status is a proxy for L1 activity in a given tissue or cell type. Indeed, it is not surprising that host cells do not rely on just one mechanism to protect themselves against uncontrolled retrotransposition. Instead, they depend on a battery of redundant defence mechanisms. In sum, this timely study provides an essential finding in our understanding of the multilayered machinery that is needed to keep TEs in embryonic development under control and enforce genome stability: TET proteins can now be seen as key players in both TE activation and repression.

## Abbreviations

5caC: 5-carboxycytosine
5fC: 5-formylcytosine
5hmC: 5-hydroxymethylcytosine
5mC: 5-methylcytosine
BER: Base excision repair
ESC: Embryonic stem cell
KRAB-ZFP: Krüppel-associated box domain-containing zinc finger protein
L1: Long Interspersed Element 1
LTR: Long terminal repeat
TE: Transposable element
TET: Ten-eleven translocation
